# Ultrastructure and Function of the Male Reproductive System in the Rotifer *Asplanchnopus* cf. *multiceps* (Rotifera: Monogononta)

**DOI:** 10.1002/jmor.70150

**Published:** 2026-07-27

**Authors:** Thiago Quintão Araújo, Robert L. Wallace, Elizabeth J. Walsh, Rick Hochberg

**Affiliations:** ^1^ Department of Biological Sciences University of Massachusetts Lowell Lowell Massachusetts USA; ^2^ Department of Biology Ripon College Ripon Wisconsin USA; ^3^ Department of Biological Sciences University of Texas at El Paso El Paso Texas USA

**Keywords:** copulation, penis, sperm, testis, zooplankton

## Abstract

Male rotifers are both rare and inconspicuous members of freshwater environments. Consequently, their anatomy is poorly known, and many details about sexual reproduction remain vague. Here, we explore the reproductive anatomy of the dwarf male of *Asplanchnopus* cf. *multiceps* using light microscopy, confocal microscopy, and transmission electron microscopy to describe its fine structure and function and compare it to what is known among other male rotifers. We reveal a male system consisting of a single testis and vas deferens, two prostate glands, and several muscles that function for copulation. The testis is comprised of a thin cellular lining that surrounds spindle‐shaped spermatozoa and tapered rods with an ultrastructure similar to those in *Asplanchna*. Both are released from the testis via a junctional sphincter, which permits their entry into a ciliated vas deferens. The vas deferens consists of three hollow cells, the most distal of which is a copulatory cell that projects into a partly ciliated male canal. During copulation, retractor muscles dilate the canal opening before a semicircular muscle contract to increase hydrostatic pressure at the posterior end. We hypothesize this pressure forces secretions from the prostate glands out the opening and just prior to canal evagination. The hollow copulatory cell at the base of the vas deferens is centered in this evagination and together creates a bulbous copulatory organ that projects from the rotifer's posterior end. This copulatory cell has microvilli that may help to form a strong bond with the prostatic secretions on the female integument. Longitudinal muscles along the vas deferens contract and pressurize the vessel, thereby ejecting rods and spermatozoa through the female integument. Additional muscles function to retract the entire complex back into the body. The organization of the reproductive system is similar to males of *Asplanchna* and this study expands on what is known from historical light microscopical analyses.

## Graphical abstract

Ultrastructure and function of the male reproductive system in the rotifer Asplanchnopus cf. multiceps (Rotifera: Monogononta). Thiago Q. Araújo*, Elizabeth Walsh, Robert L. Wallace, Rick Hochberg. The fine structure of the dwarf male of *Asplanchnopus* cf. *multiceps* possesses a singular testis with testicular rods and spermatozoa, ciliated vas deferens, and copulatory organ. Specific muscles extend the copulatory organ through hydrostatic pressure, revealing a bulbous organ comprised of a central microvillar cell (terminal end of the vas deferens) surrounded by two prostate glands and epithelia of the male canal. Fine muscles of the vas deferens discharge rods and spermatozoa into the female partner.

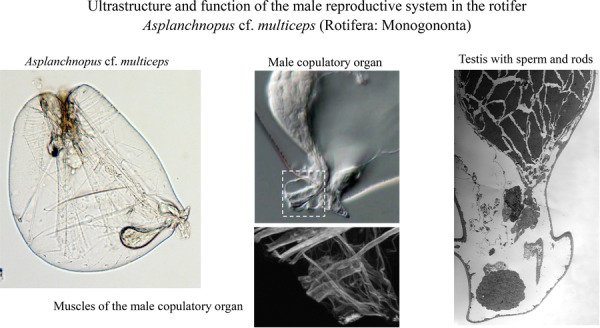

## 1 Introduction

Rotifers are widely regarded as some of the most important ciliated micrometazoans in freshwater ecosystems because of their key roles in the microbial loop and animal food webs (Wallace 2002; Wallace et al. 2015). These roles are dominated by female monogonont rotifers, which are cyclical parthenogens that can reproduce rapidly by amictic diploid parthenogenesis (Gilbert 1993). As cyclical parthenogens, amictic females occasionally produce sexual (mictic) eggs under certain environmental conditions and/or population‐level triggers (e.g., Gilbert 2003, 2004; Colinas et al. 2023). These females have meiotic ovaries (germaria) that produce haploid ova, which if unfertilized, develop into haploid males. Haploid males mate with sexual females to produce diapause embryos (resting eggs) that eventually hatch into diploid parthenogenetic females (reviewed in Gilbert 1988, 1989, 1992). To date, most studies of organ system anatomy in rotifers are confined to parthenogenetic females, but despite their importance in maintaining heterozygosity in a population, there are relatively few details on the fine structure of males (Aloia and Moretti 1973a, 1973b, 1974; Clément and Wurdak 1991).

Male rotifers may be morphologically similar to or markedly different from their female counterparts. In some benthic species, males possess a relatively complex morphology, including a developed corona, a complete digestive tract with mastax and trophi (jaws), and a foot, although males are still somewhat smaller than their female counterparts (Wesenberg‐Lund 1923; Remane 1929; Riemann and Kieneke 2008; Fontaneto and De Smet 2015). In contrast, planktonic males are typically dwarfs and may be substantially smaller than females (sometimes less than one‐third of the female body length). These males are often morphologically dissimilar, with a simplified corona and a reduced or degenerate digestive tract lacking a mastax, and in some species the digestive system is entirely absent; males in these species never feed during their short life cycles (Ricci and Melone 1998). Their extremely small size and short life spans are likely the reasons for the near absence of detailed ultrastructural studies of male organ systems.

To date, there are few ultrastructural investigations of male reproductive organs (Koehler 1965; Aloia and Moretti 1973a, 1973b) and all from a single genus (*Asplanchna*). This is surprising considering that early light microscopical research suggested that male organs might be extremely diverse despite their miniscule size. The most comprehensive light microscopical study of male reproductive morphology was conducted by Wesenberg‐Lund (1923), who examined more than 50 species and proposed a classification of males based on the structure of the penis. According to his observations, males may possess: (1) an invaginated hyaline tube functioning as a penis, (2) an invaginated cup‐shaped penis, (3) an external fold of skin that becomes erect through internal body pressure, (4) an external and permanently erect penis, or (5) a posterior body region that functions as a surrogate penis. This diversity of genital structures is intriguing because rotifers do not engage in intromission, and females lack a vagina that would permit it. Instead, the male attaches to the female and releases rod‐shaped secretions that presumably perforate the female integument, allowing sperm entry through hypodermic impregnation (Koehler and Birky 1966; Aloia and Moretti 1973a, 1973b; reviewed in Gilbert 1983b, 1988; Fontaneto and De Smet 2015).

Here we provide an integrative description of the male reproductive system of *Asplanchnopus* cf. *multiceps* using light microscopy, confocal laser scanning microscopy, and transmission electron microscopy (TEM). Our goals were to (1) describe the fine structure of the system, (2) determine how copulation occurs, (3) compare it to knowledge of male *Asplanchna*, and (4) decide how the system fits into one of the five categories described by Wesenberg‐Lund (1923).

## 2 Methods

### 2.1 Sampling and Extraction Methods

Plankton samples were collected with a 63‐μm mesh net from Flint Pond, a freshwater pond in Massachusetts, USA (42°40′30.4“N, 71°25′32.9“W)71 from July through September 2023. Samples were placed in small open buckets and returned to the University of Massachusetts Lowell at ambient temperature. In the laboratory, small volumes of the pond water were decanted into Petri dishes (90 x 15 mm) and examined using Zeiss® Stemi stereomicroscopes. Specimens of female *Asplanchnopus* cf. *multiceps* were removed and placed in a small glass bowl (5 mL) and observed while swimming. Some laid eggs that hatched as males. Other males were captured in the plankton nets.

### 2.2 Light Microscopy

Copulation between a male and a female was captured with a Sony Handycam digital camera mounted on a Zeiss stereomicroscope. Some specimens were placed in glass bowls with 1 mL of carbonated water for anaesthetization. Specimens were then transferred to glass microscope slides and examined at high magnification with a Zeiss Axio A1 compound microscope equipped with DIC. Digital photomicrographs were taken using a JENOPTIK GRYPHAX® AVIOR camera, and digital images were processed in JENOPTIK GRYPHAX® image analysis software.

### 2.3 Confocal Laser Scanning Microscopy (CLSM)

Five specimens were anesthetized and fixed in 4% paraformaldehyde in 0.1 M PBS for 2–24 h for cytochemical staining of muscular cells. Specimens were rinsed (3 × 20 min) in 0.1 M PBT (pH 7.2) and transferred to ActinGreenTM 488 ReadyProbesTM reagent (Invitrogen, Thermo Fisher Scientific) for 1.5 h at room temperature. Specimens were next rinsed in PBS for 5 min and mounted in Vectashield with DAPI (VECTOR LABORATORIES, Burlingame, USA) on glass slides and refrigerated at 4° C for at least 24 h before examination. Whole‐mount specimens were examined on a Leica TCS SP8 LSCM confocal microscope at the University of Massachusetts, Lowell. For confocal microscopy, Leica Application Suite X (LAS X) was used to collect a series of optical sections at 0.05 μm/slice. Confocal z‐stacks were collected and processed as LIF files. The saved files were imported and processed in Imaris x64 (v. 9.9.0) to render 3D images and create JPEG photos of the projections. No manipulations of the original images were made other than changes of color (false coloring or grayscale) and cropping.

### 2.4 TEM

Two specimens were prepared for electron microscopy (TEM). Both specimens were anesthetized with carbonated water and preserved in 2.5% glutaraldehyde in 0.1 M sodium cacodylate buffer (pH 7.3) for 48 h. Preserved specimens were rinsed in buffer (pH 7.3) for 1 h, postfixed in 1% OsO_4_ for 1 h, rinsed again in buffer, and dehydrated through a series of alcohols (50%, 70%, 90%, 95%) for 15 min and then 100% for 30 min (twice). Both specimens were then processed through a series of ethanol: Spurr's low‐viscosity resin in proportions of 3:1 (4 h), 1:1 (20 h), and 1:3 (4 h) on a slow rotator at room temperature. Specimens were next transferred to pure resin (2 h) and then embedded in pure resin in 00 BEEM capsules ® for 24 h in a 60°C oven. Blocks were trimmed with a razor blade and serially sectioned on a Leica UC7® ultramicrotome with a diamond knife (DIATOME ®). One male was serially sectioned in the frontal plane and one in the sagittal plane. Both males were partly contracted: one had the corona fully withdrawn, and the second male had it partly contracted. The posterior ends of both males were also partially contracted. All sections were collected on gold grids. Grids were stained with Uranyless (2 min) and lead citrate (2 min) and examined on a Philips CM10® TEM at 80 kV and equipped with a side‐mounted Gatan Orius® digital camera. Measurements of cells and organelles were taken using Fiji (ImageJ, v2.1.0/1.53c) and include number of cells or organelles examined across multiple sections (*n*) and includes averages plus/minus one standard deviation. Digital photographs were edited using Adobe Photoshop® (Release 22.5, 1990–2021), and schematic drawings were created using Adobe Illustrator®.

## 3 Results

### 3.1 Behavior and Gross Anatomy

**Figure 1:**
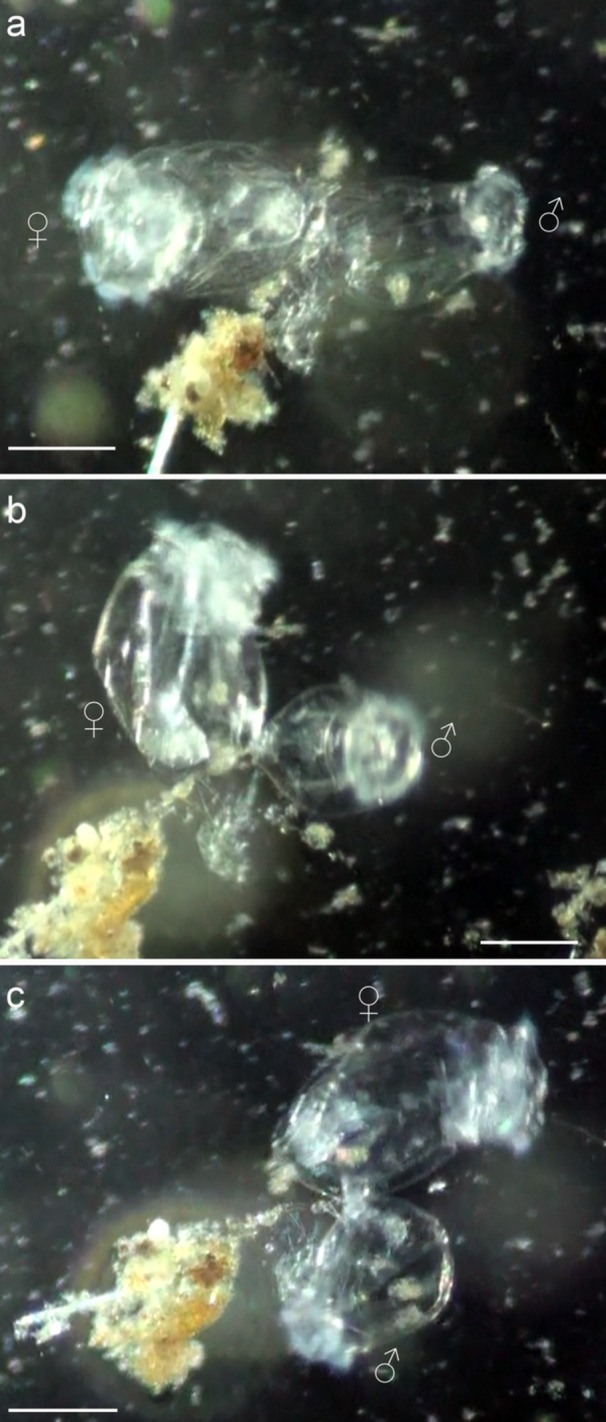
Still images from a video of copulation between a male and female of *Asplanchnopus* cf. *multiceps*. The video (Supplementary file) reveals the mating pair spinning around their axis of attachment. Scale bars: (a–c) 150 μm.

**Figure 2:**
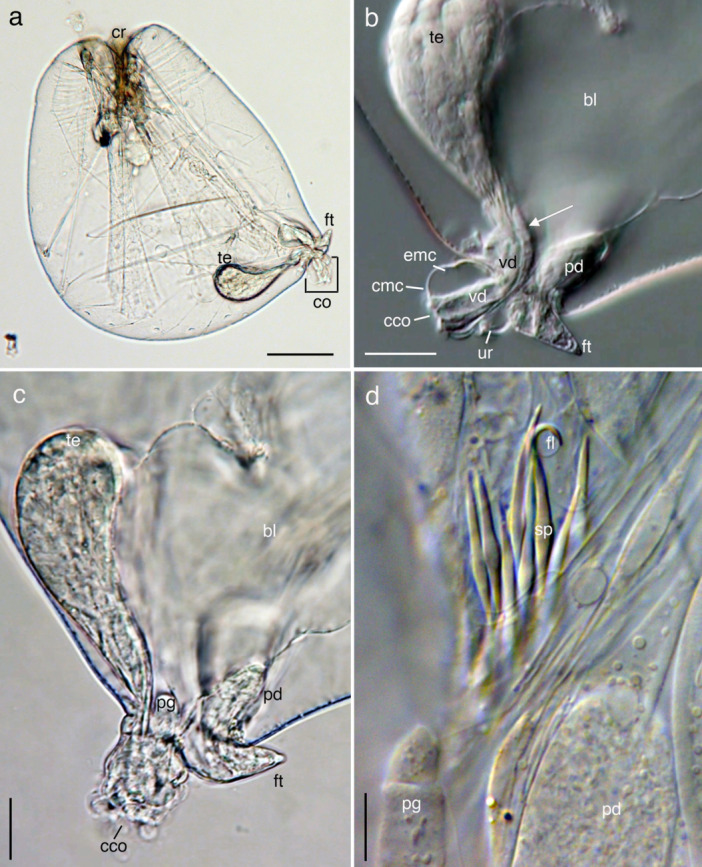
Light microscopy of live male *Asplanchnopus* cf. *multiceps*. (a) Full body with corona contracted and copulatory organ extended. (b) Differential interference contrast of the male reproductive system. The arrow points to an accessory cell of undetermined function. (c) Brightfield image of male anatomy revealing a prostate gland not observed in panel c. (d) Close‐up of the testis showing spermatozoa. Abbreviations: bld, bladder; cco, copulatory cell opening; cmc, evaginated, ciliated male canal epithelium; co, copulatory organ; cr, (retracted) corona; emc, evaginated male canal epithelium; fl, curled flagellum of spermatozoon; ft, foot; pd, pedal gland; pg, prostate gland; sp, spermatozoa; te, testis; te, testis; ur, region of the urethra opening. Scale bars: (a) 50 μm; (b) 20 μm; (c) 20 μm; (d) 10 μm.

Prior to mating, only a portion of the reproductive system could be observed. It consisted of a single testis, a ciliated vas deferens, and a short canal that terminated in an invaginated epidermis. The testis had an inverted teardrop shape, a thin cellular lining, and contained spermatozoa that appeared refractile with brightfield microscopy. Spermatozoa had spindle‐shaped cell bodies that were 15.6–21.7 μm long ($\overset{®}{x}$ = 19 ± 2.3 μm; *n* = 6) (Figure 2d). We did not measure the length of the flagellum. We did not observe rods in our live specimens. When in copula, a portion of the male system was extended from the male's body and attached to the female (Figures 1, 2). Both rotifers rotated around this axis of attachment while in copula (Figure 1; also Supp. 1). The testis moved back and forth during copulation. After separation, a copulatory “organ” (Figure 2a, “co”) remained extended from the male's body for a period. This organ was ca. 30 μm long, bulbous, and consisted of a partly ciliated epithelium (Figure 2b) surrounding the terminal end of the vas deferens (Figure 2b,c). A pair of prostate glands was also present (Figure 2c,d). An additional pair of smaller accessory cells of unknown function was present closer to the posterior body wall (arrow, Figure 2b).

### 3.2 Muscles Involved in Reproduction

The male reproductive system was closely associated with muscles in longitudinal, oblique, semicircular, and circular orientations. Many of these muscles inserted on tissues of the male system, while others appeared to parallel the testis and vas deferens and insert on the posterior integument.

**Figure 3:**
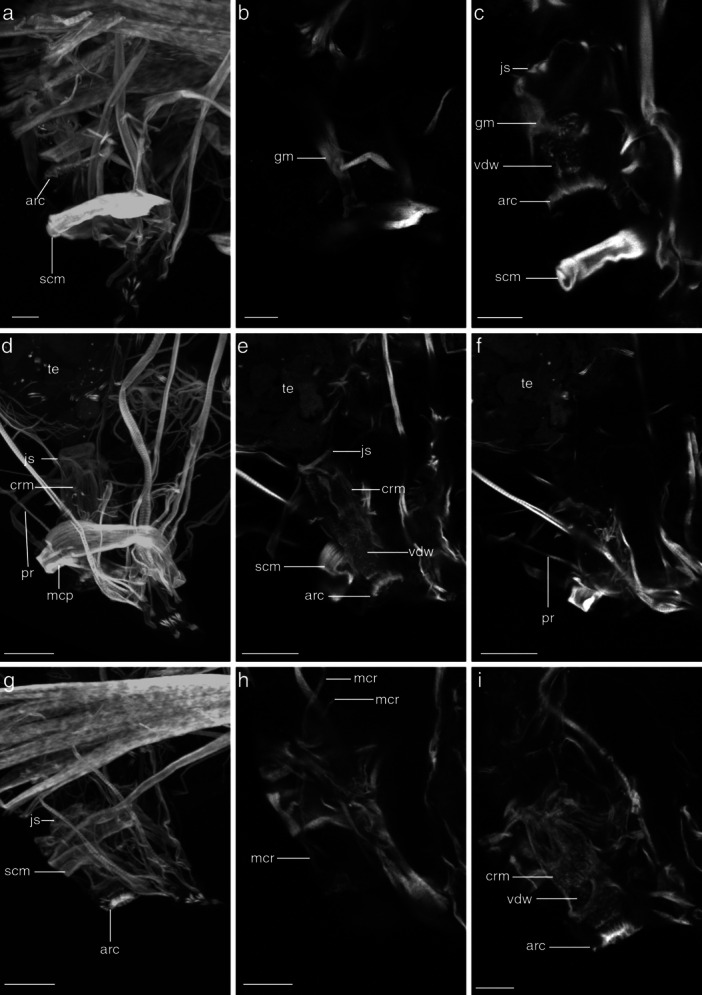
Confocal laser scanning micrographs of the muscles around the male reproductive system of *Asplanchnopus* cf. *multiceps*, lateral view. (a, d, g)—maximum intensity projection of the whole apparatus. (b, c, e, f, h, i)—Confocal z‐stack projections. (a–c): male copulatory organ fully retracted; (d–f): male copulatory organ slightly retracted; (g–i): male copulatory organ protracted. Abbreviations: arc, actin‐rich area at the tip of the copulatory cell; crm, male canal retractors; gm, genitocutaneous muscle; js, junctional sphincter; mcr, copulatory retractor muscles; pr, posterior retractors; scm, semicircular protractor; te, testis; vdw, epithelium of the vas deferens. Scale bars: (a) 8 μm; (b) 10 μm; (c) 10 μm; (d) 20 μm; (e) 20 μm; (f) 20 μm; (g) 20 μm; (h) 20 μm; (i) 10 μm.

There was a single circular muscle (*junctional sphincter*: js) at the junction between the testis and vas deferens (Figure 3c,d,e,g). The muscle was approximately 8 μm thick. This muscle appeared to be the origin of several other muscles. One muscle (*genitocutaneous muscle:* gm) originated from the sphincter and extended obliquely to the lateral body wall, where it inserted on the integument (Figure 3b,c). Several very thin (0.5 μm) longitudinal muscles (*copulatory retractor muscles:* crm) extended posteriorly from the junctional sphincter and formed a sleeve‐like arrangement around the entire perimeter of the vas deferens (Figure 3d,e,i). Each muscle appeared to insert on or near the male canal just posterior of the copulatory organ. Internally, the vas deferens epithelium was weakly stained with phalloidin and so may be composed of a strong actin cytoskeleton (Figure 3i). The distal end of the vas deferens had a very strong phalloidin signal and so may be actin‐rich (arc, Figure 3i).

Five somatic muscles were present near the reproductive system but were not directly connected to any reproductive organs (Figure 3). A pair of thin (2 μm) longitudinal muscles (posterior retractors: pr) originated from a dorsal region in the corona and inserted at the posterior end: their contractions shaped an invagination of the posterior end dorsal to the foot (Figure 3d,f). A second pair of thin (1 μm) longitudinal muscles (male canal retractors: mcr) originated from the left and right sides of the body—at approximately mid‐body length—and inserted into the male canal (Figure 3h). These muscles were only observed when the copulatory apparatus was protracted outside the body. A single semicircular muscle (semicircular protractor*:* scm) extended from the ventral side of the body, bent around the male complex and male canal retractors at approximately the testis‐vas deferens junction, and reinserted back on the ventral body wall (Figure 3a,c,e,g). In live specimens, contraction of this muscle appeared to pinch the posterior body region below the testis and extend the copulatory organ.

### 3.3 Ultrastructure of Testis and Gametes

**Figure 4:**
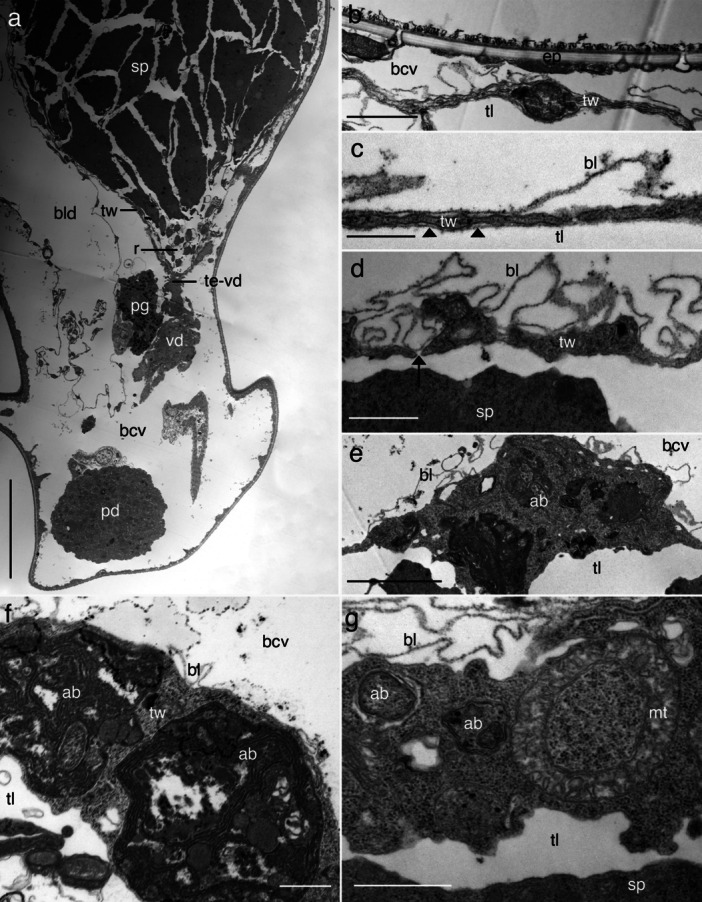
Ultrastructure of the testis in male *Asplanchnopus* cf. *multiceps*. (a) Section revealing most of the testis and posterior portions of the male reproductive anatomy. (b). Close‐up of the testis cellular lining, which is proximal to the body wall in this section. (c) Close‐up of the basal lamina on the outside of the testis lining and a very thin glycocalyx‐like layer on the luminal side of the lining (arrowheads). (d) Close‐up of the wavy basal lamina on the outside of the testis lining. Arrow shows a gap in the cellular lining. (e) Large autophagic body in the testis cellular lining. (f) A pair of large autophagic bodies in the testis cellular lining. (g) Close‐up of the testis lining showing donut‐shaped mitochondria and small autophagic bodies. ab, autophagic bodies; bcv, body cavity; bl, basal lamina; bld, bladder; ep, epidermis; pd, pedal gland; pg, prostate gland; sp, spermatozoa; te‐vd, testis‐vas deferens junction; tl, testis lumen; tw, testis cellular lining. Scale bars: (a) 25 μm; (b) 1.2 μm; (c) 500 nm; (d) 500 nm; (e) 1 μm; (f) 1 μm.

A single testis took up most of the body cavity below the degenerate stomach and next to the bladder (Figure 4a). Muscles in various orientations were present outside the testis, but none appeared to insert on the testis cellular lining. The lining was loosely covered by a highly folded basal lamina of 27–154 nm ($\overset{®}{x}$ = 68 ± 48 nm; *n* = 12) across most of its surface (Figure 4b–f). The lining lacked cell borders, that is, appeared syncytial, and was 89–531 nm thick ($\overset{®}{x}$ = 275 ± 164 nm; *n* = 8): the extreme variation in thickness was due to presence of large autophagic bodies that added substantial width to an otherwise very thin cellular lining (Figure 4e,f). There was at least one region where the lining appeared incomplete (arrow, Figure 4d). The cytoplasm was generally electron‐opaque due to a high abundance of ribosomes. Only a single nucleus was observed around the perimeter of the entire testis lining. Rough endoplasmic reticulum (rER), Golgi, membrane‐bound secretory vesicles, and mitochondria were present, though all were sparsely distributed. Some mitochondria were ring‐ or donut‐shaped (Figure 4g). Autophagic bodies were abundant and diverse in size and contents: many contained rER, electron‐dense membranes, and vesicles of various sizes and shapes (Figure 4f). The luminal side of the cellular lining had a glycocalyx‐like appearance that sometimes formed a fuzzy lamina that was tightly appressed to the surface (arrowheads, Figure 4c). Unidentified muscles were often closely affiliated with the cellular lining but never formed obvious cell junctions.

**Figure 5:**
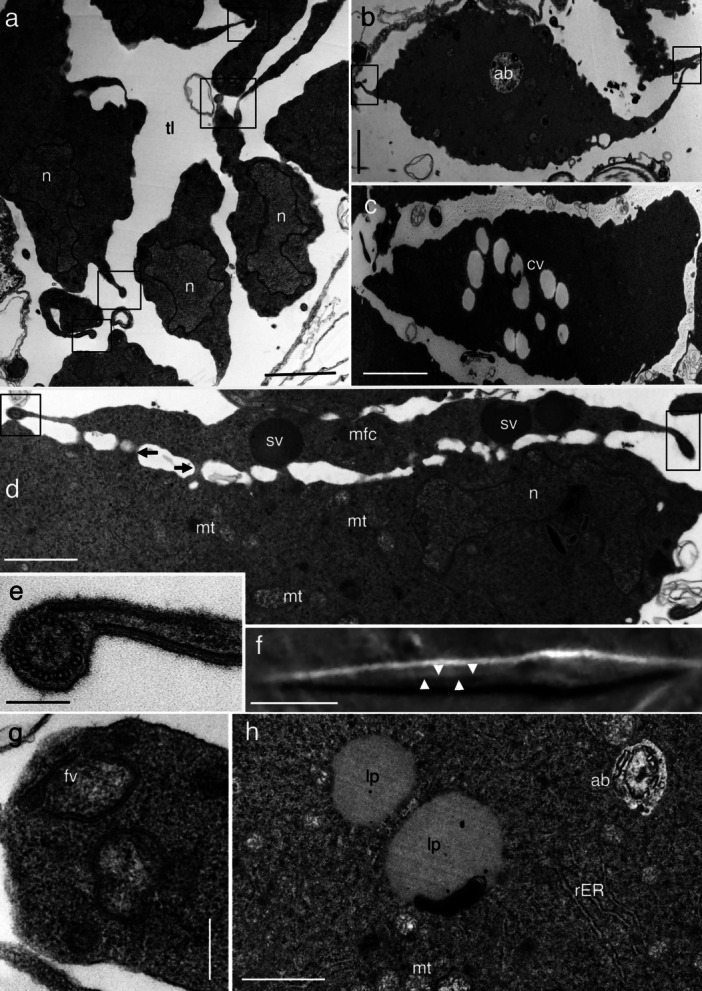
Structure of the spermatozoa of *Asplanchnopus* cf. *multiceps*. Boxes outline cross sections of the flagellar axoneme in panels (a, b, and c). (a) Three spermatozoa with polymorphic nuclei. (b) A spermatid with axoneme profiles at either end of the cell. (c) Spermatozoa with clear vesicles. (d) Longitudinal section of a spermatozoon showing the membrane flagellar complex. (e) Cross‐section of the axoneme of a spermatozoon flagellum. (f) Light micrograph of a single spermatozoon showing the longitudinal ridge (white arrowheads) that defines where the flagellum is beneath the cell's plasma membrane. Free flagellum not in view. (g) Close‐up of fenestrated bodies in a spermatozoon. (h) Close‐up of a spermatozoon showing lipid droplets and other organelles. ab, autophagic bodies; cv, clear electron‐lucent vesicles; fv, fenestrated vesicles; lp, lipid droplets; mfc, membrane flagellar complex; mt, mitochondria; n, nucleus; rER, rough endoplasmic reticulum; sv, secretory vesicles. Scale bars: (a) 1.7 μm; (b) 1 μm; (c) 4 μm; (d) 800 nm; (e) 180 nm; (f) 5 μm; (g) 400 nm; (h) 500 nm.

The testis was divided into two generic regions based on contents: most of the volume, above the junction to the vas deferens contained spermatids (immature), spermatozoa (mature), and occasional rods; posterior of this region contained atypical (rod‐secreting) germ cells and free rods. Spermatozoa had an irregular pyriform shape (Figure 5a) and a single polymorphic nucleus (Figure 5a,c; $\overset{®}{x}$ = 3675 ± 550 nm; size range: 2178–4125 nm; *n* = 7) with a well‐defined electron‐dense nuclear membrane. Chromatin was always condensed toward the periphery of the nuclear membrane (Figure 5a,d). Most sections revealed sperm with a single cross‐section of a flagellum at one end (Figure 5a), while others showed cells with cross‐sections at both ends (Figure 5b): the first are considered spermatozoa and the second spermatids (Koehler 1965) (Koehler 1965). Long sections revealed the flagellum‐undulating membrane complex *sensu* Melone and Ferraguti (1999): a membrane extended along one side of the cell and contained the flagellar axoneme (seen in cross section at both ends of the cell: Figure 5d). All axonemes contained a 9 × 2 + 2 axoneme (Figure 5e). The cytoplasm of the cell body and flagellum undulating membrane complex was electron‐dense and contained electron‐dense secretions. Only the cell body contained lipids, mitochondria, rER, and polymorphic vesicles reminiscent of autophagic bodies ($\overset{®}{x}$ = 322 ± 95 nm; size range: 159–447 nm; *n* = 7). Autophagic bodies had distinct plasma membranes and included various contents, including smaller vesicles and membranes in an opaque cytosol (see Figure 5b). Some spermatozoa had electron‐lucent vesicles present (Figure 5c). Others had vesicles with electron‐dense membranes that appeared fenestrated (Figure 5g). Many had large lipid droplets (Figure 5h).

**Figure 6:**
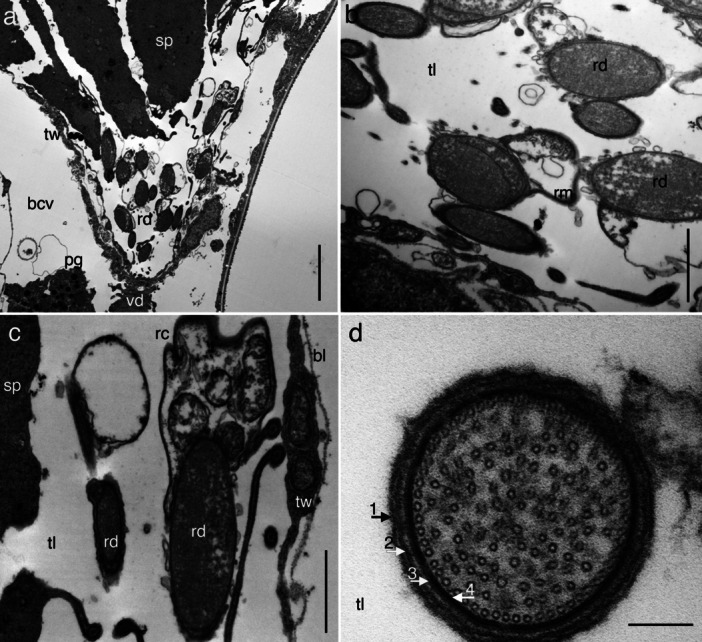
Ultrastructure of the testicular rods of *Asplanchnopus* cf. *multiceps*. (a) Longitudinal section revealing the presence of rods at the base of the testis and near the junction with the vas deferens. (b) Cross sections through individual rods, some of which still possess a membrane derived from the rod‐producing cells in the testis. (c) Rod still attached to a rod‐producing cell in the testis. (d) Cross‐section through a single immature rod to reveal the three membranes (1–3) and gaps between membranes that surround the rod. The innermost electron‐dense membrane (4) is the only membrane that remains after other membranes have been shed. Inside the rod lumen are filaments, revealed to be hollow in section. bcv, body cavity; bl, basal lamina; pg, prostate gland; rc, rod‐secreting cell in partial section; rd, testicular rod; rm, electron‐dense rod membranes that appear to be shed during rod maturation; sp, spermatozoa; tl, testis lumen; tw, testis cellular lining; vd, vas deferens. Scale bars: (a) 5 μm; (b) 600 nm; (c) 1.2 mm; (d) 110 nm.

Rods were present mostly toward the base of the testis and close to the vas deferens (Figure 6a). Rods were spindle‐shaped based on partial longitudinal sections and generally circular in cross‐section (Figure 6a). In longitudinal section, the rods often possessed one or two electron‐dense membranes (22–35 nm thick; $\overset{®}{x}$ = 29 ± 4 nm; *n* = 11) around an inner electron‐lucent core of longitudinally oriented fibers (Figure 6b,c). Rods were tapered, and so measurements of diameters depended on where the section was taken. Maximum diameter (presumed mid‐region of rods) was relatively consistent (998–1113 nm thick; $\overset{®}{x}$ = 1005 ± 45 nm; *n* = 11) in both specimens. While most rods possessed one to two walls around the inner core, some rods had 3–4 wall‐like layers (Figure 6d). Most walls were separated from each other by an electron‐lucent space. Walls were always electron dense. We provide measurements of a single rod in near perfect cross section that had four walls and three with intervening spaces: first (outermost) wall of 21 to 34 nm thick ($\overset{®}{x}$ = 28 ± 4 nm; *n* = 10); electron‐lucent space of 18 to 29 nm wide ($\overset{®}{x}$ = 25 ± 5 nm; *n* = 10); second wall of 24 to 40 nm thick ($\overset{®}{x}$ = 31 ± nm; *n* = 10); space of 18 to 36 nm wide ($\overset{®}{x}$ = 29 ± 5 nm; *n* = 10); third wall of 22 to 33 nm thick ($\overset{®}{x}$ = 28 ± 3 nm; *n* = 10); and no space separating the third wall from the innermost wall of 9–13 nm thickness ($\overset{®}{x}$ = 11 ± 1 nm; *n* = 10). The innermost wall was the only one with a distinct trilaminar appearance. The internal fibers of the rod (and all rods) were dispersed around the periphery of the rod lumen and appeared to be perfectly aligned next to the inner rod wall (Figure 6d). Approximately 60 fibers (in cross sections) were present around the periphery of most rods: the fibers themselves were circular in cross section with an electron‐dense perimeter and a hollow core: diameters were 11–15 nm ($\overset{®}{x}$ = 14 ± 1 nm; *n* = 26).

### 3.4 Ultrastructure of Vas Deferens and Posterior Canal

**Figure 7:**
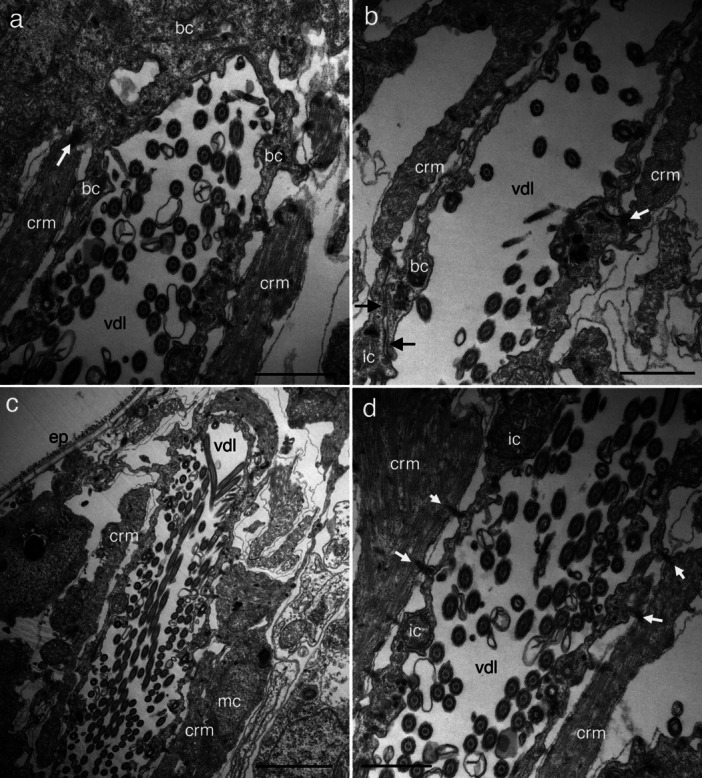
Ultrastructure of the vas deferens of *Asplanchnopus* cf. *multiceps*. (a) Oblique section of a bend in the vas deferens. The top of the image shows a tangential section through the vas deferens, while the remainder of the vas deferens is observed in longitudinal section. The lumen contains many cilia, and muscles line the outside of the epithelium. (b) Longitudinal section through the vas deferens to reveal the copulatory retractors on the outside of the epithelium. Black arrows show a junction between two cells of the vas deferens epithelium. (c) Section showing more of the vas deferens (separate specimen) and the ciliated lumen; (d) Close‐up of the spot desmosomes (white arrows) that connect the copulatory retractor muscles to the vas deferens epithelium. bc, basal cell of the vas deferens; crm, copulatory retractor muscles; ep, epidermis; ic, intermediate cell of the vas deferens; mc, muscle cell nucleus; vdl, lumen of the deferens. Scale bars: (a) 2 μm; (b) 1.5 μm; (c) 3.5 μm; (d) 2 μm.

The region below the testis was composed of three zones made from at least five interconnected cells that formed a continuous, hollow, ciliated canal: the vas deferens (three cells: basal cel, intermediate cell, and copulatory cell), a ciliated male canal cell, and an invagination of the posterior body wall (two epidermal cells). The vas deferens was bent in our specimens, and so provided both tangential and longitudinal views. It consisted of two three cells that extended from the base of the testis to the ciliated posterior canal. The anterior cell, herein called the basal cell (following the terminology of Aloia and Moretti 1973b) extended posteriorly from the testis lining (Figure 7a,b). We could not determine whether this cell was a direct extension of the testis lining or was a separate cell, but presence of cilia in the hollow of this cell (and absent from the testis lining) suggested they are different cells (Figure 7a,b). Compared to the testis lining, the basal cell possessed a slightly more opaque cytoplasm containing few nuclei, sparse mitochondria, unidentified membranes, and many ribosomes (Figure 7). Ciliary rootlets were not observed. Septate desmosomes connected the basal cell to the more posterior intermediate cell (black arrows, Figure 7b). The intermediate cell possessed many of the same organelles, including cilia, but also had autophagic bodies (not shown). Both cells had numerous muscle junctions (spot desmosomes) along their length (white arrows, Figure 7d). The distal end of the intermediate cell where it connected to the copulatory cell had a more electron‐dense cytoplasm that corresponded to the actin‐rich zone observed in CLSM (see Figure 3i).

**Figure 8:**
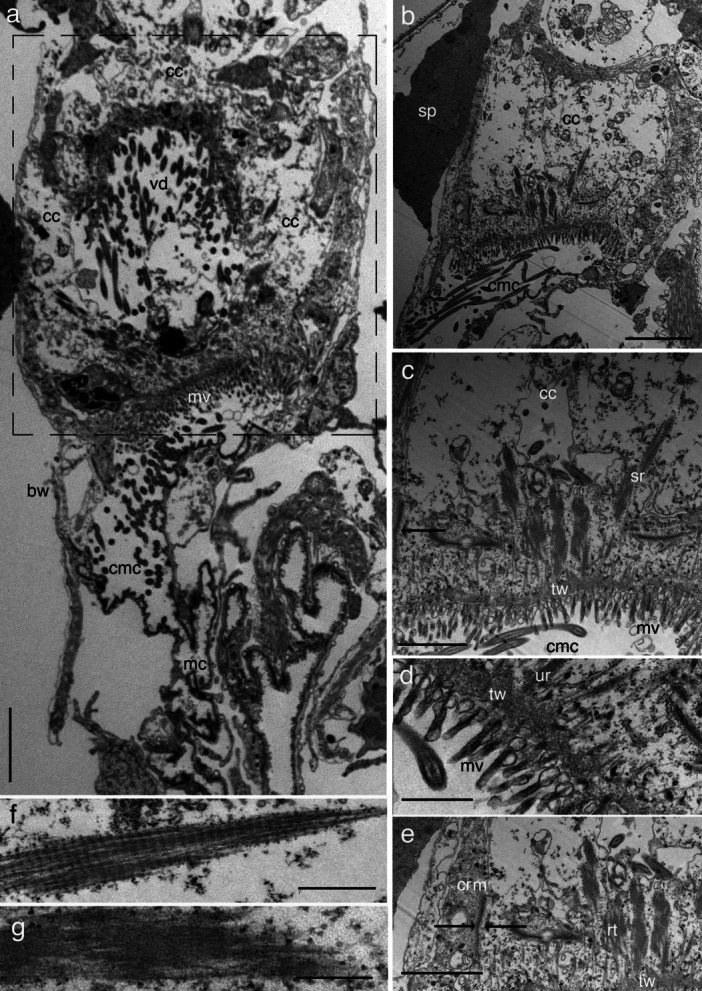
Ultrastructure of the copulatory organ (copulatory cell plus male canal and invaginated epidermis). (a) Longitudinal section through the copulatory organ. The copulatory cell is surrounded by a dashed box line. The vas deferens (in cross‐section) enters the cell at an angle. (b) Longitudinal section through the copulatory cell, revealing the terminal end covered with microvilli. A spermatozoon is on the outside of the cell (the reason for which is unknown). (c) Close‐up of the terminal end of the copulatory cell. Ciliary rootlets are distributed throughout the cytoplasm. (d) Magnified view of the microvilli and actin‐rich terminal web beneath them. (e) Septate desmosome (black arrows) connecting the copulatory cell to a copulatory retractor. (f) Section of a striated rootlet. (g) Section of an unstriated rootlet near the microvilli. bw, body wall; cc, copulatory cell; cmc, (invaginated) ciliated male canal; crm, copulatory retractor muscle; mc, (invaginated) male canal; mv, microvilli; sr, striated rootlet; tw, terminal web; ur, unstriated rootlet; vd, vas deferens. Scale bars: (a) 2.5 μm; (b) 2.5 μm; (c) 2 μm; (d) 500 nm; (e) 2 μm, (f) 250 nm; (g) 200 nm.

The intermediate cell joined the distal copulatory cell at an angle in our specimens, i.e., the intermediate cell was somewhat perpendicular to the copulatory cell in all sections (Figure 7a). We could not determine if the intermediate and copulatory cells joined end‐to‐end or in some other fashion: e.g., the copulatory cell projected into the intermediate cell as in *Asplanchna* (see Aloia and Moretti 1973b). The copulatory cell body was cylindrical, hollow, and approximately 8.5 μm wide by 10 μm long: this is likely an underestimate due to planes of section (Figure 8a). The cell had an electron‐lucent cytoplasm, a single nucleus, small patches of ribosomes, few mitochondria, several non‐striated fibrous rootlet‐like structures, and a few striated rootlets throughout the cytosol (Figure 8). The tip of the cell was covered in numerous microvilli (608–798 nm long, $\bar{x}$ = 707 ± 61 nm; *n* = 23) that extended from its rim (Figure 8a–d). Just interior to the microvilli was a dense terminal web of 201–353 nm thickness ($\bar{x}$ = 274 ± 60 nm; *n* = 11) (Figure 8c,d). Longitudinal sections of non‐striated rootlet‐like fibers were present near the microvilli (ur, Figure 8c,g). Striated ciliary rootlets were mostly organized in an a:p orientation and present throughout the cytoplasm (sr, Figure 8c,f). Longitudinal copulatory retractor muscles were present around the cell perimeter and connected to it via septate desmosomes (Figure 8e). These same muscles extended posteriorly to the male canal.

**Figure 9:**
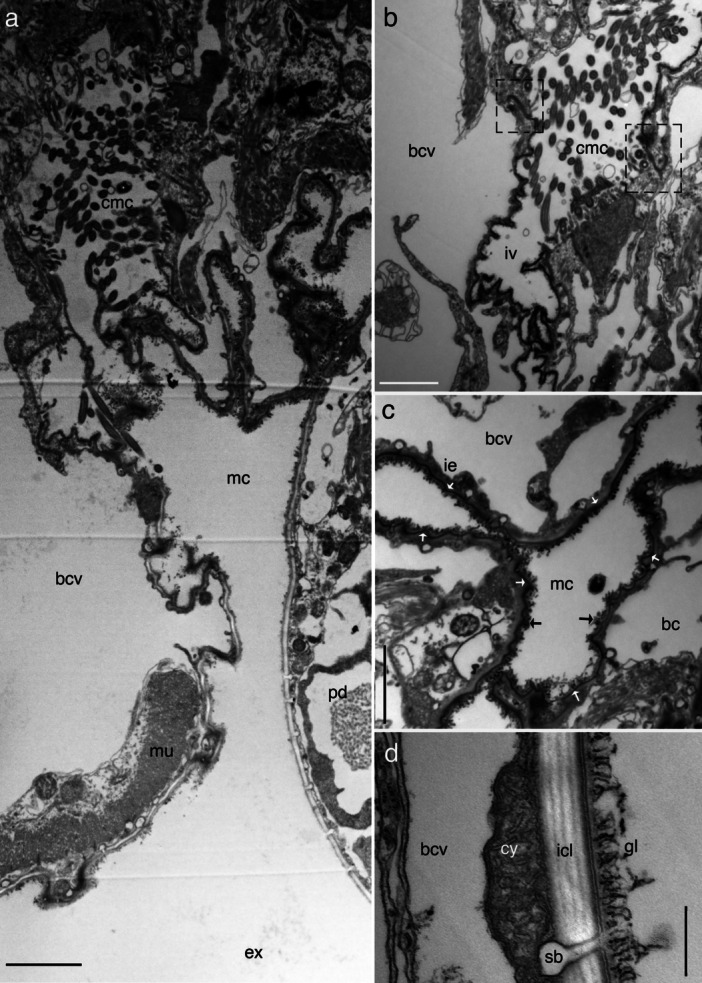
Ultrastructure of the canals posterior of the copulatory cell. (a) Longitudinal section revealing the interconnected ciliated male canal and invaginated epidermis that forms a temporary, posterior canal. (b) Close‐up of the ciliated male canal cell where it joins the invaginated epidermis. Boxed regions show septate desmosomes that connect the two epithelia. (c) Close‐up of the highly folded invaginated epidermis. Black arrows point to the glycocalyx‐like layer on the inside of the canal. White arrows point to the very thin and electron‐dense intracytoplasmic lamina inside the epidermis. (d) Ultrastructure of the epidermis that lines the body for comparison to the invaginated integument. bcv, body cavity; cmc, (invaginated) ciliated male canal; cy, cytoplasmic region of the epidermis below the intracytoplasmic lamina; ex, external environment; gl, glycocalyx; icl, intracytoplasmic lamina of the epidermis; iv, invaginated male canal; mc, (invaginated) male canal; mc, ciliated male canal; mu, muscle; pd, pedal gland; b, secretory bulb in the epidermis. Scale bars: (a) 5 μm; (b) 800 nm; (c) 1.2 μm; (d) 500 nm.

The copulatory cell projected into a ciliated male canal (mc, Figure 9a–c). The canal cell had septate desmosomes (ca. 1.4 μm long) along its length where it connected to copulatory retractors muscles. The ciliated canal cell formed desmosomes with an invaginated region of the posterior integument (iv, Figure 9b). The invaginated epidermis appeared to consist of two epithelia in a:p sequence. They could be discerned from the more anterior canal cell because they lacked cilia, possessed a very thin and electron‐dense intracytoplasmic lamina (ICL, 39–77 nm thick; $\bar{x}$ = 55 ± 12 nm; *n* = 9; white arrows, Figure 9c), and contained a glycocalyx‐like layer on the luminal side (black arrows, Figure 9c). This glycocalyx‐like layer also covered the body epidermis (icl, Figure 9d). The body epidermis was thicker (243–805 nm; $\bar{x}$ = 426 ± 177 nm; *n* = 10) than the epithelium of the invagination (188–394 nm; $\bar{x}$ = 256 ± 90 nm; *n* = 9), and the ICL had additional layers (Figure 9d).

### 3.5 Ultrastructure of Prostate Glands

**Figure 10:**
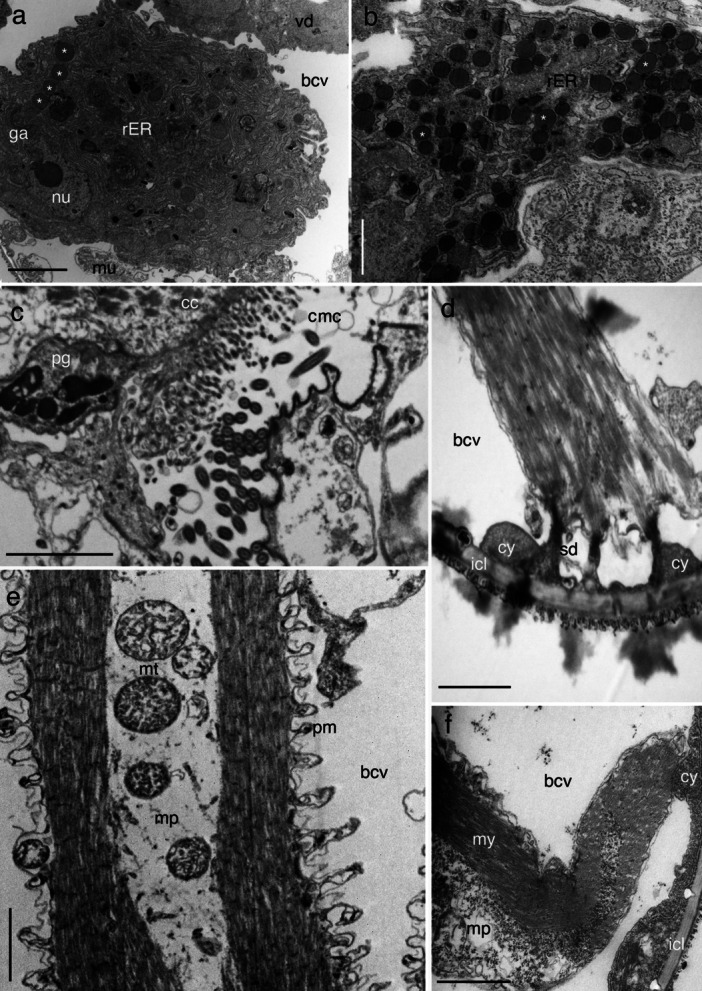
Ultrastructure of the prostate glands and some muscles that play a role in copulation in *Asplanchnopus* cf. *multiceps*. (a) Section through one of the two prostate glands revealing extensive rough endoplasmic reticulum and a series of small electron‐dense secretory vesicles (*). (b) Section through a second prostate glands to reveal the numerous electron‐dense secretions. (c) Section showing the terminal region of a prostate gland next to the copulatory cell. (d) Longitudinal section through a posterior retractor muscle revealing a wavy plasma membrane surrounding a pair of thick myofiber regions and a central core of myoplasm and organelles. Z‐rods are regularly arranged down the length of the muscle. (e) Close‐up of a posterior retractor muscle where it joins the body wall. Electron‐dense spot desmosomes connect the two. (f) Section through the semicircular protractor muscle where it joins (cell junction not shown) the body wall. Z‐rods are not obviously present in this muscle. bcv, body cavity; cc, copulatory cell; cmc, ciliated male canal; cy, cytoplasmic region of the syncytial integument; ga, Golgi apparatus; icl, intracytoplasmic lamina of the syncytial integument; mc, male canal; my, myofibers; mp, myoplasm; muscle; pg, prostate gland; rER, rough endoplasmic reticulum; vd, vas deferens. Scale bars: (a) 2 μm; (b) 1.5 μm; (c) 2 μm; (d) 1.2 μm; (e) 1 μm; (f) 2 μm.

Gland bodies were present on either side of the vas deferens. Both glands possessed a tightly adpressed basal lamina around their plasmalemmae: the laminae were 66 to 141 nm thick ($\bar{x}$ = 100 ± 34 nm; *N* = 14). Each gland contained an electron‐dense cytosol filled with abundant ribosomes, numerous mitochondria, voluminous rER cisternae, few scattered Golgi cisternae, and one to two polymorphic nuclei (3610–4024 nm maximum diameter) (Figure 10a,b). Secretions were often centered in the glands, and all possessed an electron‐dense membrane surrounding a homogeneous electron‐dense core (Figure 10b). Secretions in both glands had similar size ranges (total range: 174–553 nm in diameter, $\bar{x}$ = 422 ± 67 nm; *N* = 24), and their electron densities were similar. The glands appeared to open in the ciliated male canal (Figure 10c). The process of secretory vesicle production and exocytosis were not documented (but see Aloia and Moretti [1973b] for details on *Asplanchna brightwellii* Gosse, 1850).

### 3.6 Ultrastructure of Muscles Involved in Copulation

All muscles had highly folded basal laminae covering their plasma membrane, possessed oblique striation, and had dense bodies (60–100 nm; $\bar{x}$ = 70 ± 10 nm) distributed throughout the length of the cells (Figure 10c,d). The only muscles that could be identified with some certainty in TEM were the copulatory retractors (Figure 7), posterior retractors (Figure 10c,d), and semicircular muscle (Figure 10f). Nuclei, mitochondria, and other organelles were generally centralized within the myoplasm between bundles of myofilaments (Figure 10c) or more rarely to their periphery (Figure 10e). Most longitudinally oriented muscles, except the copulatory retractors, appeared weakly cross striated: the z‐rods were regularly spaced (450–640 nm; $\bar{x}$ = 567 ± 88 nm; *n* = 15) inside the myofibers (Figure 10c): these patterns could not be detected with brightfield or DIC optics. This arrangement was not observed in the *semicircular sphincter* (Figure 10e). The posterior retractor muscles that inserted on the posterior integument did so through multiple macula adherens junctions. These junctions were always electron dense and consisted of bands of densely staining filaments in both the muscle cell myoplasm and the cytoplasm of the integument (Figure 10d). Small gaps could be observed between the muscle and integument cell junctions (range 30–40 nm$,\bar{x}$ = 33 ± 5 nm; *N* = 10; not shown).

## 4 Discussion

### 4.1 Male Anatomy

Most knowledge of rotifer reproductive anatomy is based largely on light microscopical observations (reviewed in Gilbert 1983a, 1983b, 1988, 1989, 1992, 1993; see also Fontaneto and De Smet 2015). Relative to females, male rotifers are known for their relatively “simple” anatomy. Males possess a singular testis that connects to a vas deferens (ductus seminalis of Wesenberg‐Lund [1923]) and a copulatory “organ”, often considered the penis (Wesenberg‐Lund 1923). Various prostate glands are affiliated with the male system and may function in attachment to the female during copulation. Accessory cells of unknown function may also be present (Wesenberg‐Lund 1923). In the absence of a formal vagina, sex occurs when a male attaches to a female and inseminates her through her body wall, the location of which is species‐ and context‐dependent (Rico‐Martínez and Snell 1997; Alvarado‐Flores et al. 2017). Insemination requires the male to first discharge small testicular rods through the female's integument, thereby creating an entry point for spermatozoa to follow: muscles of the male system are presumably responsible for the discharge of rods and gametes into the female.

Wesenberg‐Lund (1923) provided the most detailed descriptions of male anatomy to date, with later reviews by various authors (Remane 1929; Hyman 1951; Gilbert 1983b; Fontaneto and De Smet 2015). In Wesenberg‐Lund's analyses, he described several types of penis (aka copulatory organ) from across Class Monogononta. The work is impressive, but notably, the author never provided a strict definition for the term “penis,” which complicates our understanding of how the male system works and how it evolved. In this seminal work, the author described several categories of penis‐like organ: (1) an invaginated hyaline tube (e.g., species of Epiphanes Ehrenberg, 1832); (2) an invaginated cup‐shaped organ (e.g., species of *Asplanchna*); (3) an external fold of skin that can be made erect from internal body pressure (e.g., species of Brachionidae); (4) an external and continually erect organ (some species of Brachionidae (formerly described as species Anaeuridae)); and (5) a posterior body region that functions as a surrogate penis (e.g., species of Polyarthra Ehrenberg, 1834). Importantly, Wesenberg‐Lund implies there is a correlation between the length and structure of the vas deferens and the existence of a “true” penis, but never explicitly states what a true penis is. We suspect that a true penis in Wesenberg‐Lund's reasoning is one that is “forced out of the genital opening” (1923, 138) to deliver spermatozoa but also functions for excretion (more below). Examples from Wesenberg‐Lund's study include the copulatory organs of species of Brachionus, Cephalodella Ehrenberg, 1830 (formerly *Diglena*), and *Trichocerna* Lamarck, 1801 (formerly *Rattulus*): all have cup‐shaped penes when extended beyond the body wall. Other species are described to possess a ductus seminalis that turns inside out during extension; these are not considered true penes: for example, *Epiphanes senta* (Müller, 1773) and most species of Notommatidae (Wesenberg‐Lund 1923). Whether or not species of *Asplanchnopus* are also among the species with a “true” penis remains unknown, though *A. multiceps* (originally described as *A. myremeleo* in Wesenberg‐Lund 1923) is described to have a protrusible penis (Wesenberg‐Lund 1923, 66). In our study, we analyzed specimens of *A*. cf. *multiceps* using brightfield microscopy, confocal laser scanning microscopy, and TEM to gain better anatomical and functional insights into the structure of the male system and determine if an how it fits into one of Wesenberg‐Lund's (1923) categories.

### 4.2 Fine Structure and Function of the Male System

At the ultrastructural level, the male system of *A*. cf. *multiceps* is like that of species of *Asplanchna* (Koehler 1965; Aloia and Moretti 1973a, 1973b). In *Asplanchna*, the testis is described to have a thin cellular lining with a few nuclei and no cell junctions other than where it connects to the vas deferens. Similarly, the testis lining of *A*. cf. *multiceps* a thin and appears syncytial. A basal lamina lines the testis and separates it from muscles and the bladder, and a thin glycocalyx‐like layer is pressed tightly against the luminal side of the testis. Numerous organelles are distributed around the periphery of the cellular lining of the testis, especially autophagic bodies, but in general, no organelles are especially abundant.

Sperm shape and ultrastructure are like what has been described in other monogononts including species of *Asplanchna* (Aloia and Moretti 1973b, 1974; Koehler 1965). The spermatozoa are spindle‐shaped cells with a long flagellum at one end. They have a polymorphic nucleus, occasional dense bodies (secretions), clear vesicles, lipid droplets, mitochondria, and a flagellum‐undulating membrane complex with an internal flagellum that projects from the anterior end (Koehler 1965) (Figure 2d). Sectional profiles of some sperm reveal a flagellum at both ends of the sperm: these are considered spermatids by Koehler (1965). Light microscopical measurements of mature spermatozoa body show they have an equivalent size (15.6–21.7 μm long) to the spermatozoa of *Asplanchna* (15–20 μm long: Melone and Ferraguti 1999) but are longer than those of *E. senta* (8–10 μm long: Melone and Ferraguti 1999) and shorter than those of *Brachionus plicatilis* Müller, 1786 (30–35 μm long: Melone and Ferraguti 1994, 1999). Like the spermatozoa of *Asplanchna*, mitochondria are abundant and widely dispersed throughout the cell body: in *Brachionus*, the mitochondria are concentrated in the postnuclear region of the cell (Melone and Ferraguti 1994, 1999). Fenestrated vesicles are present in some spermatozoa and are reminiscent of the fenestrated bodies/donut‐shaped vesicles of *Asplanchna* (Koehler 1965).

Rods are present at the base of the testis in our preserved specimens, but were not observed in the live specimens. At the ultrastructural level, the rods had a comparable organization to those in other monogononts: they have a circular profile, one or more membranes on the outside, and a cavity full of tubules (Koehler and Birky 1966). We did not attempt to follow the secretion and maturation of the rods as has been done in studies of *Asplanchna* (Koehler and Birky 1966), but our observations are in line with those of *Asplanchna*: (1) the rods are produced from atypical germ cells; (2) many rods possess multiple outer membranes that are likely indicative of being immature, that is, they have not yet shed the outer membranes derived from the Golgi (Koehler and Birky 1966); and (3) mature rods have only a single outer membrane. All rods were 500 nm to 1 μm in diameter and contained hollow filaments of 11–15 nm diameter. The only other data on internal rod filaments comes from the study of *Asplanchna* sp., where filament diameters were 20 nm (Koehler and Birky 1966). These cross‐sectional dimensions correspond in size to microtubules (e.g., tubulin: Wade 2009), as well as intermediate filaments such as keratin and lamins (Herrmann and Aebi 2004). An antibody‐based confocal analysis would be a useful future study to identify specific proteins that make up these tubules.

The testis leads to a vas deferens, which is constructed of three cells interconnected by desmosomes, like the condition described for *A. brightwellii* (Aloia and Moretti 1973b). The first two cells that form a hollow channel from the testis—the basal and intermediate cells—possess cilia that project into their shared lumen (Figures 12b; 13b). The cilia presumably function to move rods and gametes down the vas deferens. A circular muscle (*junctional sphincter*) at the top of the vas deferens may function to regulate their passage. The distal end of the vas deferens is formed from a single cell—the copulatory cell—which is hollow, shorter than the first two cells, and bears microvilli on its distal surface. The microvilli are underlain by a thick terminal web of actin (stained with phalloidin) and numerous fibrous roots. Curiously, the cell cytoplasm contained several striated rootlets, but no cilia could be observed projecting from the microvillar end of the cell. However, there were numerous profiles of cilia in the canal below; some of these may be from the copulatory cell, but others are products of the canal cell. Regardless, the copulatory cell projects into a ciliated male canal that itself joins an invaginated region of the posterior epidermis we call the male canal.

**Figure 11:**
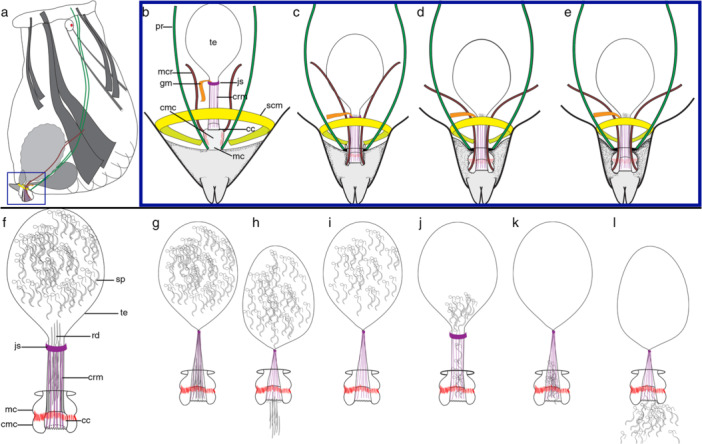
Schematic of the male reproductive system of *Asplanchnopus* cf. *multiceps* with a hypothesis for extension of the copulatory organ and the ejection of rods and gametes. (a) Lateral view of the male. Somatic muscles in dark gray; foot, pedal gland, and testis are light gray; muscles involved in copulation are in different colors. (b–e) Dorsal view of the copulatory organ during extension. Note that the male canal is formed from invaginated epidermis and is partly ciliated. To achieve extension of the copulatory organ, one muscle pair relaxes (mcr) and two muscle pairs contract (pr, scm), leading to evagination of the male canal epithelium and the protraction of the central copulatory cell (aka the copulatory organ; see also Figures 12 and 13). (f–l) Ejection of the rods and spermatozoa happen in sequence. Relaxation of the junctional sphincter (js) allows rods to enter the vas deferens before. After its subsequent contraction, the copulatory retractor muscles (crm) of the vas deferens contract, thus shortening the vas deferens and forcing rods out the copulatory organ. Relaxation of the js and crm then allow spermatozoa into the vas deferens and the contraction‐relaxation cycle is repeated. cc, copulatory cell at base of vas deferens; cmc, ciliated male canal; crm, male canal retractors; gm, genitocutaneous muscle; js, junctional sphincter; mc, (invaginated) male canal; mcr, copulatory retractor muscles; pr, posterior retractors; scm, semicircular protractor; rd, testicular rod; sp, spermatozoa; te, testis.

Copulation appears to be dependent on muscles closely affiliated with the male system (see Figure 11). To date, the musculature of male rotifers has only been incompletely studied and never correlated with reproductive function (Wesenberg‐Lund 1923; Leasi et al. 2010), though even the earliest researchers understood that muscles play a prominent role in copulation (see references in Wesenberg‐Lund (1923). In addition to the junctional sphincter mentioned above, we observed five sets of muscles that appear to function in copulation: two posterior retractors, a single semicircular protractor, several copulatory retractors, two male canal retractors, and an oblique muscle we call the genitocutaneous retractor. These muscles are only engaged when a male attaches to a female with his foot, presumably using his pedal glands to maintain a temporary hold to her integument. However, based on live observations, CLSM, and TEM, we suspect that secretions from the prostate glands form the bond, that is, important for insemination (as described below), and as hypothesized for *A. brightwellii* by Aloia and Moretti (1973a). As they noted, the bond must be strong enough to hold the male in place while the female continues to swim.

**Figure 12:**
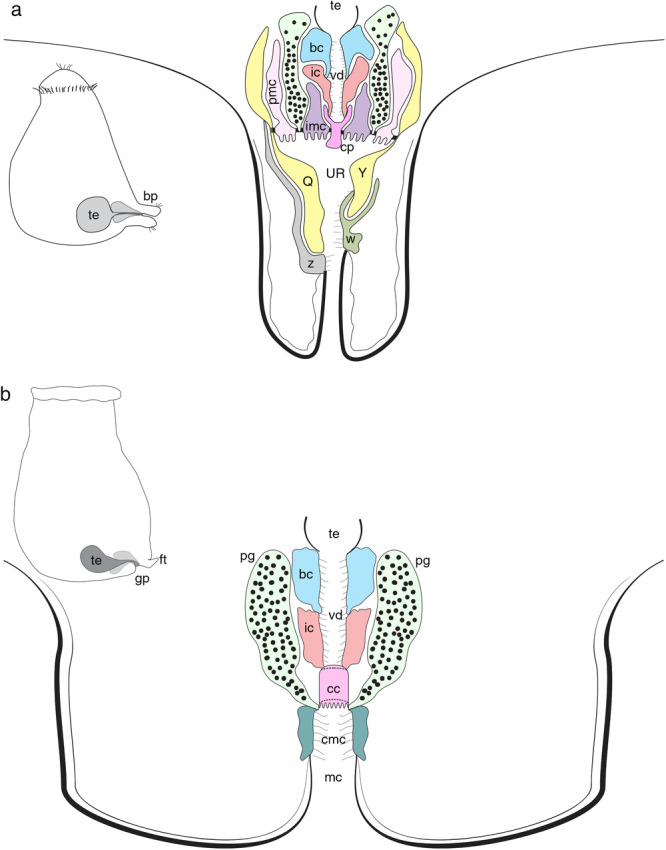
Schematic drawings of two retracted male copulatory organs viewed in longitudinal section. (a) *Asplanchna brightwellii* is redrawn from Aloia and Moretti (1973b). (b) *Asplanchnopus* cf. *multiceps*. bc, basal cell of the vas deferens; bp, body protrusion; cc, copulatory cell at base of vas deferens; cmc, (invaginated) ciliated male canal; cp, cap cell; ft, foot; ic, intermediate cell of the vas deferens; imc, internal microvillar cell; mc, (invaginated) male canal; pg, prostate gland (not all glands are shown for *A. brightwellii*); pmc, peripheral microvillar cell; ps, mucus secretion of the prostate glands; Q, Y, W, and Z are four different cell types lining the urogenital canal according to Aloia and Moretti (1973); te, testis; UR, urethral lumen; vd, vas deferens.

**Figure 13:**
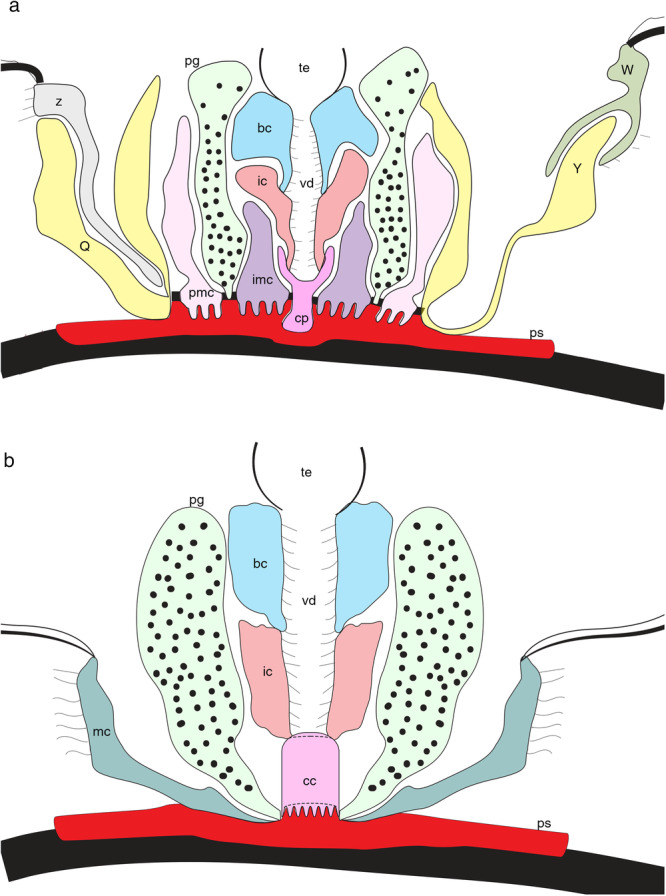
Schematic drawings of two male copulatory organs: the drawings represent hypotheses of what each organ would look like when extended outside the male body and against the female integument. (a) Interpretation of the extended organ of *Asplanchna brightwellii* based on Aloia and Moretti (1973b). (b) Interpretation of the extended organ of *Asplanchnopus* cf. *multiceps*. bc, basal cell of the vas deferens; bp, body protrusion; cc, copulatory cell; cp, cap cell; ft, foot; ic, intermediate cell of the vas deferens; imc, internal microvillar cell; mc, ciliated male canal; pg, prostate gland (not all glands are shown for *A. brightwellii*); pmc, peripheral microvillar cell; ps, mucus secretion of the prostate glands; Q, Y, W, and Z are four different cell types lining the urogenital canal according to Aloia and Moretti (1973); vd, vas deferens.

After initial attachment with his foot, a brief series of muscle contractions occur to prepare the male for insemination. (1) The posterior retractor muscles contract and pull on the posterior integument around the male canal. This canal is a semi‐permanent invagination that is maintained by tonic contractions of the paired canal retractor muscles (mcr, Figure 11), that is, the canal is present when males swim but is relaxed during copulation. We suspect that relaxation of these muscles followed by contraction of the posterior retractors dilates the posterior opening of the canal to allow for extension of the copulatory organ, which consists of the vaginated canal and base of the vas deferens (Figure 11c). (2) The semicircular protractor muscle then contracts (Figure 11c), creating a pinched region below the testis that contains portions of both prostate glands and most of the vas deferens. This pinch increases hydrostatic pressure and produces two effects in sequence. (3) It forces the prostate glands to release secretions into the canals, which are then swept by cilia out the dilated opening and onto the female integument. (4) The pressure then evaginates the male canal (consisting of both ciliated and unciliated epithelia), and in doing so, protracts the terminal end of the vas deferens; the copulatory cell is at the center of the bulbous organ that extends from the body (Figures 11c–e, 13b). The overall function of this process is to press the copulatory cell against the prostatic secretions that line the female's epidermis. The microvilli and intermicrovillous spaces of the copulatory cell should in theory provide a high surface area for the prostatic secretions to create a bond, and the underlying terminal web in the copulatory cell may reinforce the plasma membrane to prevent damage to the cell and inhibit dislodgement from the female. The presence of cilia as an outer ring around the bulbous copulatory organ is merely a result of the male ciliated canal becoming evaginated and has no obvious functional significance. In fact, cilia around “penes” of rotifers is documented from several species (e.g., Hudson 1875; Hudson and Gosse 1886; Rousselet 1897; Wesenberg‐Lund 1923; De Beauchamp 1965; Gąsiorowski et al. 2019) but its significance has never been demonstrated. We propose a similar process of canal evagination and vas deferens protraction and attachment for *A. brightwellii* based on the observations of Aloia and Moretti (1973a, 1973b) (see Figures 12, 13).

All studies of copulation have revealed that males can inseminate females in a variety of locations from the foot to the corona, presumably avoiding thickened regions of the integument that prevent penetration (Alvarado‐Flores et al. 2017). The rods of atypical germ cells are hypothesized to function in a form of dermal insemination, whereby they are forcibly ejected from the testis to create an opening in the female integument to allow for entry of spermatozoa (reviewed in Gilbert 1983b). The rods are stiff and spindle‐shaped secretions reinforced with internal tubules that presumably add rigidity, which would be necessary to avoid impacting on the surface of the female once discharged. They are also positioned at the base of the testis, which would allow them to be ejected prior to the spermatozoa. Ejection presumably occurs through muscular action, but does not appear to rely on circular muscles, which are often invoked as a means to pressurize a closed hydrostatic system (Seymour 1969; Kier 2012). The junctional sphincter at the top of the vas deferens would appear too far away to generate appropriate pressure (Figure 11f): also, contraction‐relaxation cycles of the sphincter would likely permit spermatozoa to enter the vas deferens before a hole is created in the female integument. We therefore hypothesize that this muscle only plays a role in regulating the passage of rods and (then) spermatozoa to enter the vas deferens. Another option for ejecting the rods may be through contraction of the copulatory retractors (crm, Figure 11f–h). These extremely thin longitudinal muscles originate on the junctional sphincter and terminate on the epithelia of the male canal: they connect to the vas deferens, copulatory cell, and male canal through spot and septate desmosomes along their length, suggesting their contraction moves all three tissues. In theory, contraction of these muscles should shorten the entire complex and create pressure, much like depressing a hypodermic syringe. This pressure should eject the rods. The muscles may then partly relax, but not fully so as to prevent dislodgement from the female. This should be followed by relaxation of the junctional sphincter to permit spermatozoa entry to the vas deferens. Multiple contraction‐relaxation cycles would then forcibly inject spermatozoa into the dermal pore of the female's integument (Figure 11f,j–l). Our observations of mating reveal that multiple cycles do in fact take place during insemination (see also Wesenberg‐Lund 1923).

After insemination, the male detaches from the female. We are uncertain how this occurs; we have observed detached males with extended copulatory organs (the bulbous shape remains outside the body), meaning that withdrawal of the organ is not necessary for detachment. We suspect that the prostatic secretions are only temporarily adhesive. Regardless, a male eventually retracts his copulatory organ into the body and continues swimming. This withdrawal appears to depend on several muscles: (1) relaxation of the semicircular protractor (scm), which decreases the hydrostatic pressure on the copulatory region; (2) contraction of the male canal retractors (mcr), which should effectively pull most of the entire complex inside the body once hydrostatic pressure has dissipated; and (3) relaxation of the posterior retractors (pr), which will return the integument at the posterior end to a resting (invaginated) position (Figure 11). The only muscle we are uncertain about is the genitocutaneous muscle, which has an oblique orientation as it extends from the junctional sphincter to the body wall. During contraction, this muscle should pull on the sphincter at the top of the vas deferens. This may play a role in helping to retract the complex back into the body or perhaps reorient the copulatory complex once it is withdrawn; further behavioral analyses are necessary to confirm these ideas.

### 4.3 Comparative Fine Structure of Males

Our study of the male reproductive system of *A*. cf. *multiceps* provides unique insights into its fine structure and function, but with few comparable studies of other males, it is difficult to appreciate how it fits into one of Wesenberg‐Lund (1923) categories or where it lies on the spectrum of anatomical and functional complexity. Relative to *A. brightwellii*, the only other species studied with TEM (Aloia and Moretti 1973a, 1973b), the male system of *A*. cf. *multiceps* has a similar organization but appears less complex: it consists of fewer cell types (compare Figure 12a,b) and does not appear to function as a true penis *sensu* Mammalia (Dixson 2021). However, the copulatory organ of *A. brightwellii* is a true penis in that sense: it is a protrusible organ that delivers sperm to a partner but also conveys urine to the external environment via a urogenital canal. Again, we emphasize that Wesenberg‐Lund's (1923) definition of a penis is vague. The main differences in cellular complexity between the two species may in part be related to the additional urinary function of the penis in *A. brightwellii*. In this species, there is a cap cell at the end of the vas deferens with a flap‐like extension: this extension presumably covers the opening to the vas deferens and may prevent mixing of urine and gametes (our interpretation; redrawn here as Figure 12a based on observations of Aloia and Moretti 1973b). The cap cell is surrounded by four microvillar cells (only a central microvillar cell in *A*. cf. *multiceps*) and eight prostate gland cells (two in *A*. cf. *multiceps*) whose distal ends terminate in the urogenital canal. We hypothesize that these additional cells function in copulation and not urination. We propose that *A. brightwellii* has additional prostate glands because males lack a foot for initial attachment. The additional microvillar cells are important to form a bond with these extra prostatic secretions, similar to what we propose for *A*. cf. *multiceps* (Figure 13). In total, the entire complex (minus the testis) of *A. brightwellii* consists of at least 21 cells, while that of *A*. cf. *multiceps* consists of only nine cells (based on serial reconstruction) (see Figure 12a,b).

It is clear from our analysis as well as the earlier studies of Aloia and Moretti (1973a, 1973b) that the male reproductive system is more complicated than light microscopical observations would imply. And while the dwarf body form of planktonic monogononts may appear simple, the remainder of their bodies harbor interesting complexity centered around the genital system. By comparison, the female system appears less complicated as it only contains a syncytial organ for nourishing and producing ova that are then released via an oviduct (Bentfeld 1971a, 1971b; reviews in Gilbert 1983a). There does not appear to be any special cells or muscles that contribute to its function (Reviews in Gilbert 1983a, 1989; Clément and Wurdak 1991; Kotikova et al. 2004). However, it is also clear from our study than even this might be an oversimplification because nearly all knowledge of female anatomy is based on light microscopy, which can make both the histology and associated musculature appear deceptively simple.

A recent metanalysis of sex in rotifers has revealed that many details surrounding sexual anatomy, induction of sexuality, its periodicity and frequency, and even presence of males in many species remains poorly known (Walsh et al. 2017). Importantly, without a better understanding of reproductive organs, gametes, and the behaviors that govern the sexual process—including mate guarding (Schröder 2003), mate selection (Rico‐Martinez and Snell 1995; Rico‐Martínez and Snell 1997; Gomez and Serra 1996; Joanidopoulos and Marwan 1999), and mate contact and pheromone induction (Gilbert 1963; Snell et al. 1995; Snell and Rico‐Martinez 1996; Rico‐Martínez and Snell 1997; Díaz et al. 2006; Snell et al. 2007; Snell 2011; Snell & Rico‐Martinez 2021a, 2021b)—we will continue to lack a full appreciation of the importance of sex in monogonont rotifers. For example, there is still no understanding of how dermal insemination evolved, whether or not it differs among lineages, what happens to the testicular rods after penetration, how spermatozoa make their way to the ova to initiate fertilization, and how fertilization initiates diapause egg production. These questions among many others require our attention before we can fully appreciate how efficient sexual reproduction is among species and how it affects population dynamics in different environments.

## Author Contributions

**Thiago Quintão Araújo:** conceptualization, investigation, writing – original draft, methodology, validation, visualization, writing – review and editing, formal analysis. **Robert L. Wallace:** writing – original draft, Writing – review and editing, funding acquisition, validation. **Elizabeth J. Walsh:** writing – original draft, writing – review and editing, funding acquisition, validation. **Rick Hochberg:** conceptualization, investigation, funding acquisition, writing – original draft, writing – review and editing, visualization, validation, methodology, formal analysis, project administration, supervision, resources.

## Conflicts of Interest

The authors declare no conflicts of interest.

## Data Availability

The data that support the findings of this study are available on request from the corresponding author. The data are not publicly available due to privacy or ethical restrictions. Data in the form of electron micrographs are available from the authors upon request.
